# Supplementary material for: Whole Genome Amplification in Preimplantation Genetic Testing in the Era of Massively Parallel Sequencing
Source: Int J Mol Sci. 2022 Apr 27;23(9):4819. doi: 10.3390/ijms23094819 (PMC9102663; doi:10.3390/ijms23094819)
Supplement: Supplementary file 1 [file ijms-23-04819-s001.zip › ijms-1699057-supplementary.pdf]

Supplementary Table S1. Commercial WGA kits.

**DOP-PCR**	DOPlify by Perkin Elmer			
**MDA**	REPLI-g by Qiagen	GenomiPhi V2 by Cytiva	Illustra GenomiPhi by GE Healthcare	TruePrime by Sygnis (non hexamer-based WGA)
**MALBAC**	MALBAC by Yicon Genomics	ChromInst by Yicon Genomics (integrating WGA and library preparation)		
**Ligation- mediated PCR**	Ampli1 by Silicon Biosystems			
**Hybrid methods**	PicoPlex by Takara (originally developed by Rubicon genomics)	GenomePlex by Merck (formely Sigma Aldrich)	SurePlex by Illumina	PicoSeq by Takara (PicoPlex integrating WGA and library preparation)
